# Dewatered Sludge Decorated with Nanoparticles for Alum Sludge Conditioning towards the Concept of “End-of-Waste”

**DOI:** 10.3390/nano13212903

**Published:** 2023-11-05

**Authors:** Hossam A. Nabwey, Maha A. Tony

**Affiliations:** 1 Department of Mathematics, College of Science and Humanities in Al-Kharj, Prince Sattam bin Abdulaziz University, Al-Kharj 11942, Saudi Arabia; 2 Basic Engineering Science Department, Faculty of Engineering, Menoufia University, Shebin El-Kom 32511, Egypt; dr.maha.tony@gmail.com; 3 Advanced Materials/Solar Energy and Environmental Sustainability (AMSEES) Laboratory, Faculty of Engineering, Menoufia University, Shebin El-Kom 32511, Egypt

**Keywords:** end-of-waste, aluminum-based sludge, conditioning, dewatering, magnetite, circular economy, drinking water treatment

## Abstract

The circular economy concept is leading environmental engineering in the search for “*End-of-Waste*” criteria. Untreated waste residue results from drinking water treatment plants, causing severe environmental issues, and its reuse is essential. In this regard, this investigation introduces the beneficial reuses of alum sludge cake to close the loop between sludge waste generation and reuse. Considering alum sludge as a resource for dewatering instead of its categorization as a waste reflects an “*End-of-Waste*” approach. Alum sludge cake was thermally calcined at 400 °C and named thermally treated alum sludge cake (TAS-cake). In this study, TAS-cake decorated with magnetite with a percent weight of 5 to 1%, respectively, was labeled as TAS-cake@Fe-(5-1). X-ray diffraction (XRD) and morphologies were applied to characterize the hybrid composite. A Fenton-based hybrid composite was applied to extrude water from alum sludge for 7 min of conditioning time. Furthermore, the factorial design based on response surface methodology (RSM) was applied to optimize the operational variables. TAS-cake@Fe-(5-1) and hydrogen peroxide revealed 1.2 g/L and 740 mg/L doses at pH 3.0, showing pronounced performance and revealing the highest capillary suction time (CST) reduction, which reached 53%. A temperature increase also showed a pronounced enhancement effect on the sludge dewaterability that reached 72% when 55 °C was applied. Thus, such a novel conditioner is a promising candidate for alum sludge conditioning.

## 1. Introduction

In conventional drinking waterworks plants through water purification steps, *Al* salts are applied as coagulants, leading to high concentrations of aluminum in the treatment residuals, producing so-called “*Alum Sludge*”. The result is annual waste generated around the world [1,2]. Alum sludge-based waste discharged into open land areas is a common criterion in some places. This is leading to a massive contamination of the soil and ecosystem [3]. Thus, environmental engineering and academia are searching for a solution to overcome such waste disposal criteria.

Initially, prior to sludge handling and discharge, reducing its volume via dewatering is a must. It is a necessity to reduce its volume by improving the interior dewaterability performances. The common technique is to use commercial polyelectrolytes for sludge conditioning based on organic polymers to enhance the dewatering performance [4]. In this regard, numerous sludge-conditioning techniques have been applied according to the literature. For instance, a cationic polyacrylamide stabilizer is successfully used as a conditioner [5] and polyaluminum chloride [6] as a polyelectrolyte conditioner [7]. Also, thermal treatments and sonication combined with a chemical conditioner have been investigated [8,9]. Additionally, the combination of coagulation/flocculation technology through the application of various coagulants/flocculants has been also applied [10,11]. However, the practical application needs extra research since the use of chemicals makes the process expensive, especially the use of fresh chemicals, and some other chemicals are toxic such as polyelectrolytes. Thus, advanced oxidation processes as environmentally friendly methodologies have been introduced. Therefore, selecting the appropriate conditioner is a prerequisite.

On the other hand, with ever-increasing worldwide concern, it is essential to introduce environmentally non-hazardous techniques instead of the various available toxic substances. Additionally, environmentally friendly advanced oxidation methodologies have been applied for alum sludge conditioning as an environmentally non-hazardous technique [12,13]. To date, Fenton’s reaction has been the superior conditioner for various sludge treatments [14,15].

The Fenton reaction is applied in various forms as an oxidation system and represents a promising candidate conditioner. The system is based on iron ions in incorporation with H_2_O_2_ to produce highly reactive species that oxidize the sludge particles [16,17,18,19,20]. In recent decades, the application of nanoparticles has shown superior oxidation efficiency. Such nanoscale particles include magnesium [3,20], magnesium/aluminum layered double hydroxide [19], zerovalent iron [18] and waste comprising sawdust [17]. 

However, with the fresh chemicals used, the system is still a concern, and such limitations hinder the process applications [21]. One major global consideration is the replacement of fresh chemicals using environmentally friendly materials or waste substances [22]. From this concept, examining new substances valorized from waste streams is attracting scientists’ attention [23,24]. Also, cradle-to-cradle waste administration is important for a green environment [25,26]. Thus, there is limited progress in searching for greener materials, especially for sludge management [27].

The classical Fenton system is suggested as the simplest and has been applied to treat various types of wastes, especially wastewater, with limited publications dealing with alum sludge dewatering [28,29,30]. Due to its various limitations, including sludge formation and high remnant metal ions, it has not been applied so far [31]. To overcome the system boundaries, the novelty of using the source of metals from a waste stream conjugated with an environmentally non-hazardous material is explored through the use of aluminum-based sludge [32]. This process could avoid the use of extreme chemicals since it depends on several metal ions from the waste effluent.

Herein, this study aims to introduce a novel strategy for alum sludge conditioning and dewatering performance. The investigation is based on examining a low-cost conditioner derived from waterworks plant residuals and conjugated with magnetite nanoparticles to be a source for the Fenton catalyst as an innovative conditioner. The attained composite is characterized by various techniques: XRD, SEM, EDX, FTIR and TEM analysis. The conditioner is then tested for alum sludge dewatering. The effect of working variables, such as initial composite loading, H_2_O_2_ dose, pH and operating temperature, was inspected. The optimal variables were assessed using the response surface methodology (RSM) technique using a maximum response of CST reduction efficiency.

## 2. Materials and Methods

### 2.1. Materials

Liquid aluminum-based byproduct waste named alum sludge with a moisture content of 99.59% was collected from the settling tank of the waterworks plant, located at the south part of Shebin Elkowm City in the Menoufia Governorate in the north of Egypt, where aluminum sulfate is used as a primary coagulant for treating River Nile water. The main characteristics of the obtained sludge are suspended solids of 11,850 mg/L, 286 NTU of supernatant turbidity, CST of 42 s and pH 8.5.

Also, for magnetite nanoparticles, the necessary precursors are ferrous sulfate (FeSO_4_·7H_2_O) and Ferric Sulfate Fe_2_(SO_4_)_3_, which were supplied by Qualikems Fine Chem Pvt. Ltd. (Delhi, India). Hydrogen peroxide (40%, *w*/*w*) was used to initiate the oxidation reaction. Sulfuric acid and sodium hydroxide were used for sludge pH adjustment when required. LT-25 was supplied by CIBA Specialty Chemicals and was used as the source of anionic polyelectrolyte. Additionally, Magnafloc FO-4140 supplied by SNF SAC ZACde Milieux, Andrézieux 42163, France was used as the cationic polyelectrolyte. All chemicals were of analytical gra de and used as received without further purification.

### 2.2. Synthesis of TAS-cake@Fe-(5-1) Conditioner

Initially, Fe_3_O_4_ nanoparticles were synthesized through the co-precipitation route. The process was carried out by mixing the precursors of Fe_2_(SO_4_)_3_ and Fe(SO_4_) under their stoichiometric ratios in distilled water. Then, NaOH in an aqueous solution was added in a drop-wise manner until pH reached 11.0. Thereafter, the precipitate was subjected to successive washing until the pH declined to neutral prior to drying to attain a fine powder of magnetite nanoparticles. The detailed procedure is illustrated in detail elsewhere [21].

In parallel, alum sludge powder was prepared according to the previous procedure [33,34]. Initially, the extra water content in the sludge was descanted from the sludge through gravity settling prior to air-drying, thereby resulting in a dried sludge cake containing 10% moisture content. Then, the dried sludge cakes were subjected to cleaning using distilled water to eliminate impurities. Finally, in order to reach a powder form, the sludge was subjected to oven drying and grinding through ball milling for one hour. Then, according to the preliminary work, the resultant powder was calcined in the oven for 2 h at 400 °C and labeled as TAS-cake.

The composite was attained by mixing the as-synthesized alum sludge and magnetite powder named TAS-cake @Fe_3_O_4_ in weight proportions of 5/1 ratio, labeled as TAS-cake@Fe-(5-1), and then subjected to characterization prior to use in alum sludge dewatering and a conditioning test.

### 2.3. Morphological and Structural Characterization

Characterization of the prepared TAS-cake augmented with the Fe_3_O_4_ as a composite conditioner (TAS-cake@Fe-(5-1)) was attained via diffracted X-ray diffraction (XRD) for the composite. The obtained intensity of the diffracted X-ray was recorded using XRPhillips X’pert (MPD3040, Cambridge, UK) diffractometer using Cu Ka radiation source (λ = 1.5406) run at 40 kV and 40 mA in step-scan mode of 0.02°.

Fourier-transform infrared FTIR (Jasco, FT/IR-4100, type A, MICHIGAN Ann Arbor, MI 48109 USA) was further applied to determine the type of functional group responsible for the conditioning (TAS-cake@Fe-(5-1)) by using a He-Ne laser source (λ = 632.8 nm) in KBr pellet form (0.001 g sample with 0.3 g KBr). Spectra were recorded in the 4000–350 cm^−1^ region.

Scanning electron microscopy (SEM) (SEM, Quanta FEG 250) and high-resolution transmission electron microscopy (TEM) (type Tecnai G20, FEI) were applied to investigate the morphology of the samples. Additionally, the digital TEM image was analyzed using the IMAGEJ 1.48 V program to establish the particle size distribution of the TAS-cake@Fe-(5-1) hybrid composite substance. The typically used magnifications were ×8000 and ×60,000. The main elements on the conditioner composite were assessed via the energy-dispersive spectrum (EDX).

### 2.4. Co-Conditioning Procedure

**Figure 1:**
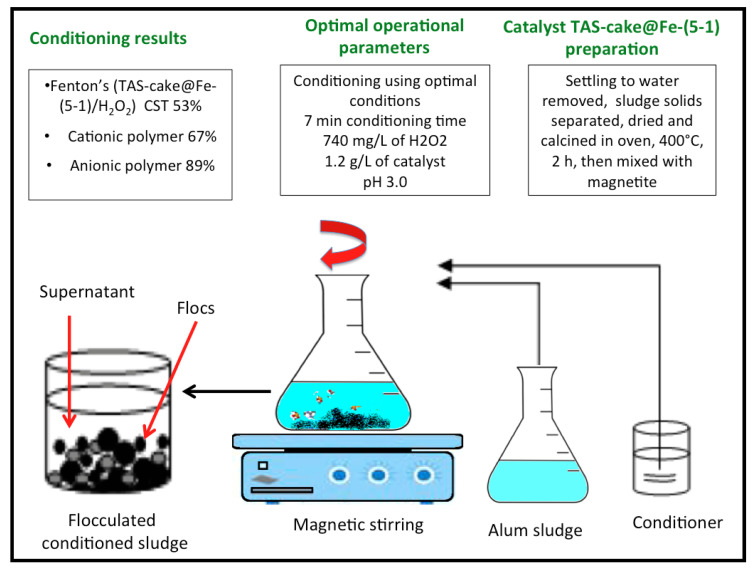
Schematic graphical representation of the experimental setup.

A jar-stirring apparatus was used to perform the conditioning tests at room temperature where blended sludge samples (100 mL) were added into a 250 mL beaker. Afterward, the pH value of the sludge was adjusted, if needed, using AD1030, Adwa instrument, Hungary. Then, the composite was added, and the oxidation reaction was initiated in the system through the addition of H_2_O_2_. Thereby, the reagents and sludge were subjected to a jar test for the specific conditioning time. Figure 1 illustrates a schematic graphical representation of the experimental setup.

The capillary suction test (CST) apparatus and SRF facility assessed the sludge dewaterability before and after co-conditioning. Triton CST apparatus (Trition-WPRL, Type 304M CST) was used for the CST measurement, and each sample was measured in triplicate. The CST reduction efficiency (%) was calculated using Equation (1).

(1) $$CST(\%)=\frac{{CST}_{o}-CST}{{CST}_{o}}\times 100$$

where CSTo and CST are the capillary suction time of the alum sludge before and after adding conditioners, respectively.

## 3. Results and Discussion

### 3.1. TAS-cake@Fe-(5-1) Composite Conditioner Characterization

#### 3.1.1. X-ray Diffraction Pattern

**Figure 2:**
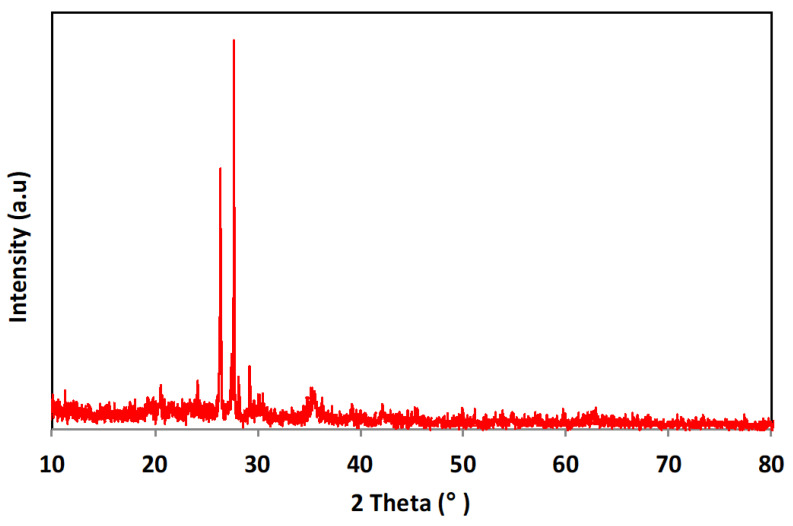
Spectrum of X-ray diffraction (XRD) for the hybrid conditioner.

Figure 2 exhibits the X-ray diffraction pattern of thermally activated dewatered sludge cake (TAS-cake) augmented with the Fe_3_O_4_ as a composite conditioner (TAS-cake@Fe-(5-1)). Notably, the crystalline nature of the composite powder comprises a sharp diffraction peak pattern. The pure cubic spine crystal structure (JCPDS No. 89-4319) of magnetite peaks is demonstrated in the sample. The XRD spectra of the peaks at 2θ values of 30.0°, 35.3°, 43.0°, 56.9° and 62.8° corresponding to the hkl of 220, 35.3°,311, 43.0° 400, 56.9°, 511 and 62.8°, 440 appear in the sample. Also, it can be seen that SiO_2_ and CaO in the (TAS-cake@Fe-(5-1)) have an amorphous structure [35,36]. Furthermore, the active substances of aluminum and graphite appear in the sample in the forms of sodium aluminum silicate (Na_1.15_Al_1.15_Si_0.85_O_4_) and calcium aluminum silicate (CaAl_2_Si_2_O_8_). This XRD spectrum verified the coexistence of Fe_3_O_4_ and TAS-cake in the hybrid composite conditioner. SiO_2_ shows the presence of peaks of 100, 101 and 110, 36. Moreover, calcium aluminum is reflected in the XRD spectrum by the peaks of 022, 112, 202 and 114. However, 002, 011 and 004 reflected the presence of sodium aluminum silicate. It is noteworthy to mention that the composite nanospheres with well-dispersed Fe_3_O_4_ nanoparticles were synthesized successfully without damaging the magnetite crystal structure using this methodology.

The crystal size of the prepared composite was also attained from this XRD pattern. The half width at half maximum was used to calculate the particle size using the Debye–Scherrer Equation (2) [1]:

D = kλ/β cosθ (2)

where D is the average diameter of nanoparticles, λ is the wavelength of X-ray radiation (1.5418 Å), θ is the diffraction angle, k = 0.9 (shape parameter) and β is the full width at half maximum of X-ray diffraction peaks. The crystal size was found to be close to 103.92 nm using the (311) index. Furthermore, the degree of crystallinity in the composite was calculated from the XRD pattern. The degree of crystallinity was calculated using the standard formula. In such a case, the crystallinity was about 54.63%, which means the prepared composite material still possessed the important properties of the crystalline material [3,16].

#### 3.1.2. Fourier-Transform Infrared Spectroscopy

**Figure 3:**
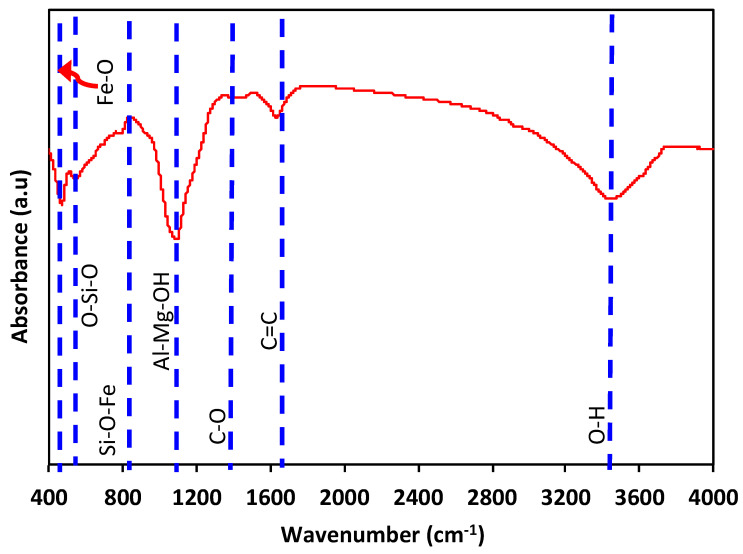
FIR spectrum of TAS-cake@Fe-(5-1) composite conditioner.

The FTIR transmittance spectrum analysis of the TAS-cake@Fe-(5-1) hybrid composite conditioner is shown in Figure 3. As illustrated in the curve in Figure 3, the bands around 420 cm^−1^ and 556 cm^−1^ are attributed to the stretching vibration of the Fe–O bonds in the sub-lattice of Fe_3_O_4_ [37,38]. The bands at 1124 cm^−1^ and 1629 cm^−1^ are a sign of the presence of C–O and C=C [39,40]. Also, the band at 440.38 cm^−1^ is allocated for the O–H stretching vibration of structural water. The Al–Mg–OH group is signified by the band at 789.7 cm^−1^ and is linked to the stretching vibrations. The bands of absorption that refer to silica appear at 467.65 cm ^−1^ of O–Si–O [23]. A summary of the bands and their corresponding peaks of TAS-cake@Fe-(5-1) composite conditioner is tabulated in Table 1. The FTIR data, Table 1 and Figure 3, of the TAS-cake@Fe-(5-1) hybrid composite conditioner estimate the presence of magnetite and Al-, Si- and C- that comprise the alum sludge cake.

#### 3.1.3. Scanning Electron Microscope Image

**Figure 4:**
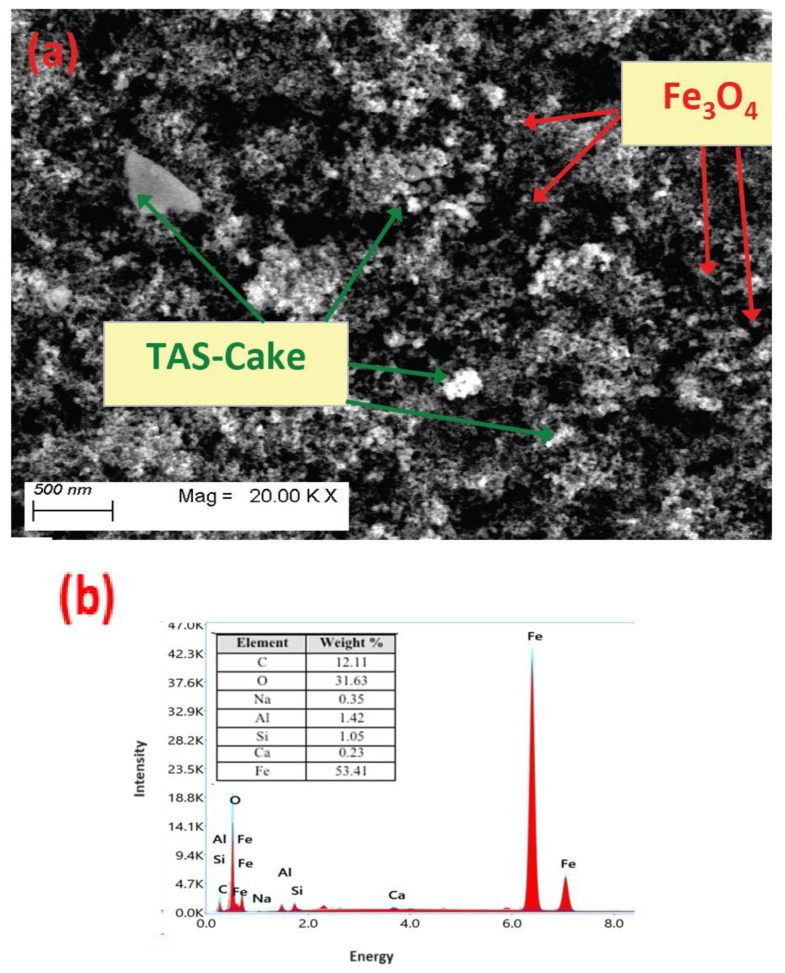
(**a**) Joint SEM images of TAS-cake@Fe-(5-1) hybrid composite conditioner and (**b**) EDX analysis report.

The SEM image was investigated to estimate and gain insight into the surface of the TAS-cake@Fe-(5-1) hybrid composite conditioner. The SEM micrograph of the modified aluminum-based sludge cake augmented with the nanoparticles of magnetite displayed in Figure 4 exhibited a semi-hexagonal sheet-like TAS-cake@Fe-(5-1) hybrid material with white spots, which reflects the presence of metals in the alum sludge cake. Such sheets are also conjugated with a spherical particle, covering the particle nanocomposite surface, which reflects the presence of magnetite. Moreover, Figure 4a displays that the sludge shape was irregular and heterogeneous with a porous texture.

The main elements in the photocatalyst samples were assessed via an energy-dispersive spectrum (EDX). EDX analysis illustrated in Figure 4b that Al, O, Si, C, Ca and Fe are the major elements of TAS-cake sludge. The TAS-cake Fe hybrid material revealed a high content of Si and Al, which could be associated with the presence of clays and aluminosilicate substances [41,42].

#### 3.1.4. Transmission Electron Microscope Image

**Figure 5:**
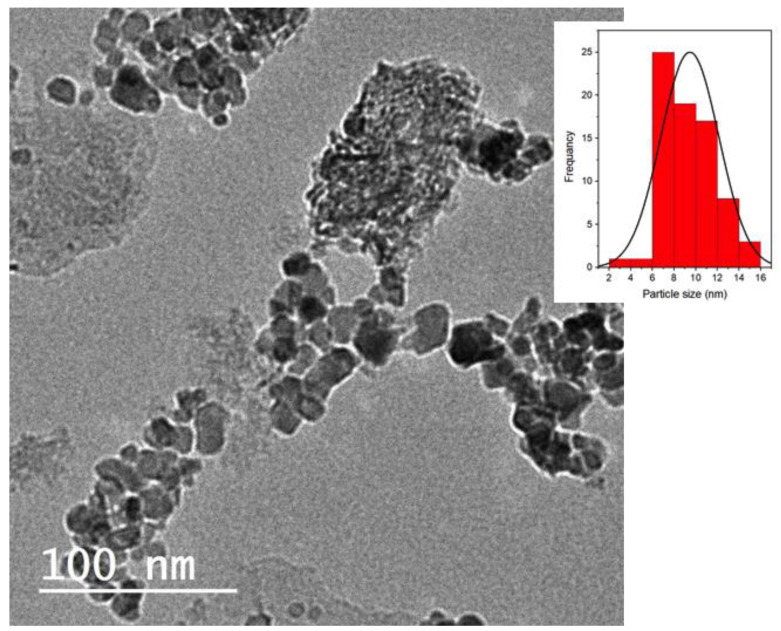
TEM micrograph of the prepared TAS-cake@Fe-(5-1) hybrid composite conditioner (inset: particle size distribution).

Figure 5 depicts TEM images of the TAS-cake@Fe-(5-1) hybrid composite conditioner. The stratified nature of the attained aluminum-based sludge is verified by the TEM image. Figure 5 representative product’s TEM pictures clearly demonstrate that the mixed hexagonal-like particles of alum sludge were augmented with spherical magnetite nanoparticles. Furthermore, very clearly visible irregular sheets signify the border of the alum sludge. Rough crystalline surfaces were attained.

Notably, the inset in Figure 5 displays the range of particle size that displays an average particle size ranging from 2 to 16 nm, which is considered a quite small particle size, demonstrating the existence of magnetite nanoparticles in the sample.

### 3.2. Aluminum-Based Sludge Dewatering Performances

#### 3.2.1. Conditioning Time Based on Hybrid Conditioner

**Figure 6:**
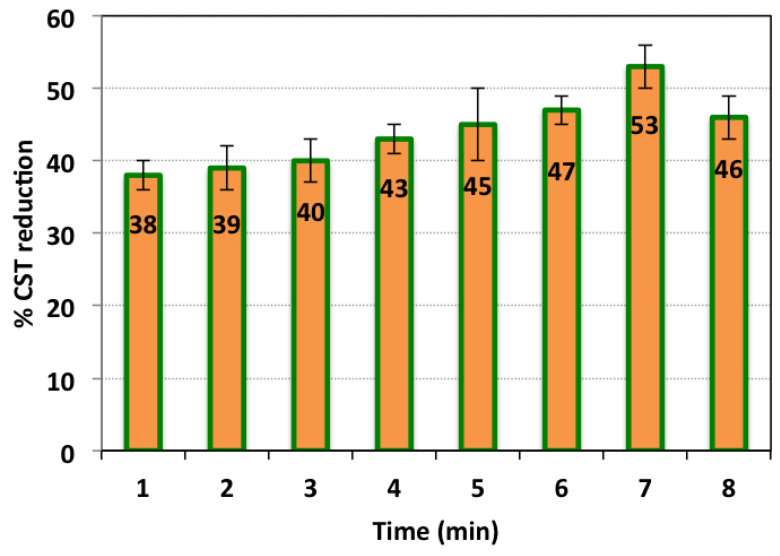
CST reduction efficiency at various conditioning times (operational conditions: pH: 3.0; TAS-cake@Fe-(5-1): 1 g/L; H_2_O_2_ dose: 800 mg/L; standard deviation of three replicates).

TAS-cake@Fe-(5-1) hybrid conditioner influence and conditioning time are jointly illustrated in Figure 6. The influence of the TAS-cake@Fe-(5-1) hybrid conditioner nanocomposite as a source of the Fenton conditioning procedure is shown according to the significance of the CST reduction. The test was carried out by changing the conditioning temperature, while all other operational parameters were kept constant, i.e., pH 3.0, TAS-cake@Fe-(5-1): 1 g/Land H_2_O_2_ dose of 800 mg/L. Commonly, minimum CST refers to a better filtration performance. The significance of the conditioning test time on alum-based sludge conditioning for better dewatering performances was assessed at different flocculation times ranging from 1 to 8 min. Various sets of experiments at various TAS-cake@Fe-(5-1) composite conditioners were checked, and the optimal conditioning time was monitored.

The results displayed in Figure 6 show that the TAS-cake@Fe-(5-1) composite augmented with hydrogen peroxide as the source of the Fenton conditioner could result in an enhancement in the CST reduction. The data showed that the influence of TAS-cake@Fe-(5-1)/H_2_O_2_ had an effective trend on the sludge conditioning and improved with the time increased. The highest CST reduction performance corresponded to a time of 7 min. However, extra conditioning time, more than 7 min, is unfavorable since the CST deduction did not improve. This investigation is in agreement with the previous research reported by Mo et al. [43].

The data obviously displayed that the maximal CST reduction (%) corresponded to a specific time of conditioning (7 min). However, with time continuation, the result is a reduction in the dewatering tendency. This might be attributed to the size of the floc formed after adding flocculent material, the main element responsible for enhancing dewaterability [44]. Also, it is necessary to mention that the sludge dewaterability enhancement by the Fenton oxidation is dependent on the release of both interstitial and chemically bound water. Interstitial water is trapped between the organics and chemically bound water or adsorbed by the oxidation and destruction of organics. It is notable that this work is in accordance with research conducted by [45] since similar results were noted with reaction time variations from 2 to 60 min. However, there was no detailed illustration of the reaction time influence on conditioning efficacy. Clearly, further work is essential to explore such an influence. Thus, TAS-cake@Fe-(5-1) is projected to be a capable alternative for substituting the traditional chemical Fenton conditioner for alum sludge. Notably, the use of waste as a recyclable substance replacing the traditional elements as a source for the Fenton reaction is a promising candidate for such a dewatering system.

#### 3.2.2. Effect of TAS-cake@Fe-(5-1) Dose

**Figure 7:**
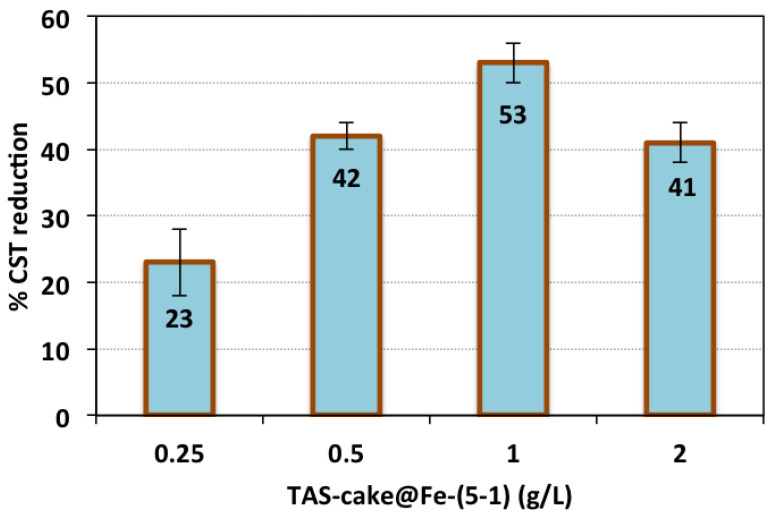
CST reduction efficiency at various TAS-cake@Fe-(5-1) doses (operational conditions: conditioning time: 7 min; pH: 3.0; H_2_O_2_ dose: 800 mg/L; and standard deviation of three replicates).

To assess the effectiveness of the TAS-cake@Fe-(5-1) on the oxidation/conditioning system as a vital role in the alum sludge conditioning, the TAS-cake@Fe-(5-1) reagent dose was altered in the Fenton system, whereas all other operating parameters were kept at the following constant conditions: conditioning time, 7 min; pH 3.0; and H_2_O_2_ dose of 800 mg/L. The results of the experiment exhibited in Figure 7 revealed that the sludge filterability regime is associated with the TAS-cake@Fe-(5-1) dose composite that is signified as a rate-limiting stage in the conditioning since its amount present in the reaction medium affects the conditioning performance.

The TAS-cake@Fe-(5-1) hybrid composite was raised from a dose of 0.25 mg/L to a dose of 1.0 g/L, and the CST decline was examined. The data plotted in Figure 7 highlighted the noteworthy role of aluminum-based sludge powder and magnetite in proceeding with the hydroxyl radicals’ generation and thereby improving the alum sludge filterability [46]. Consequently, the CST reduction increased from 23 to 53% by increasing the composite dose of the TAS-cake@Fe-(5-1) from 0.25 to 1.0 mg/L, whereas a larger amount of reagent resulted in a CST reduction, reaching only 41%.

This may be attributed to the metals in the TAS-cake@Fe-(5-1) hybrid composite, which hydrolyzed in the reaction, and their presence in optimal occurrence resulted in maximal hydroxyl radical production. This governed the oxidation reaction [47]. Also, the amount of the TAS-cake@Fe-(5-1) changed in each experiment, which means its presence affected hydroxyl radical production. Hence, the non-optimal value functions as an inhibitor of the radicals rather than a generator, thereby reducing the overall conditioning rate [48]. The OH radicals formed in the reaction media, which is associated with the presence of the catalyst as a reagent that is initiated with hydrogen peroxide and is related to the amount of catalyst added. The amount of OH radicals formed is not sufficient to attack alum sludge particles to initiate the new intermediates for enhancing the filtration properties [14].

#### 3.2.3. Effect of Hydrogen Peroxide Dose

**Figure 8:**
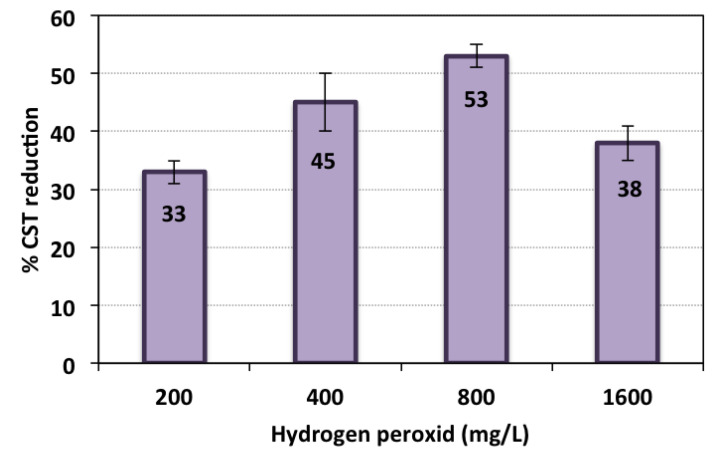
CST reduction efficiency at various hydrogen peroxide doses (operational conditions—conditioning time: 7 min; pH 3.0; TAS-cake@Fe-(5-1): 1 g/L; and standard deviation of three replicates).

The hydrogen peroxide reagent change augmented with the TAS-cake@Fe-(5-1) on the CST reduction rate is displayed in Figure 8, and other operating conditions were kept constant in the following ways: conditioning time, 7 min; pH 3.0; and TAS-cake@Fe-(5-1) 1 g/L. According to the data displayed, the increase in the hydrogen peroxide from 200 to 800 mg/L resulted in a reduction in CST, reaching 53%. Hydrogen peroxide was used as an initiator for the TAS-cake@Fe-(5-1) catalyst. However, a further upsurge in the hydrogen peroxide reagent dose reached more than 800 mg/L, and the result is a reduction in the effectiveness of CST removal. Such an impact is due to the hydrogen peroxide reagent triggering the hydroxyl radical’s production. Hydroxyl radicals are the main horsepower of conditioning through oxidation/dewatering systems. Their presence in the reaction in the optimal value leads to the maximal flocculation reaction due to the maximal hydroxyl radical generation.

The increase in the hydrogen peroxide dose improved the effectiveness of dewaterability until a certain limit of addition from the reagent. Although the enhancement in sludge dewaterability occurred at a broad range of hydrogen peroxide, reversible results of CST are attained with an extra increase in the reagent. Thus, the hydrogen peroxide reagent plays a significant role in the Fenton reaction since excess addition leads to a negative impact on the alum sludge dewatering performance. This may be linked to the amount of OH radicals, the main horsepower in the oxidation system. Hydrogen peroxide elevation leads to a specific limit of concentration; the result is a scavenging effect for the radicals produced rather than a generator. Some authors [14,21,45] in the literature reported such phenomena concerning OH radical production on the effect of the Fenton reaction, which is associated with the amount of hydrogen peroxide.

#### 3.2.4. Effect of pH Value

**Figure 9:**
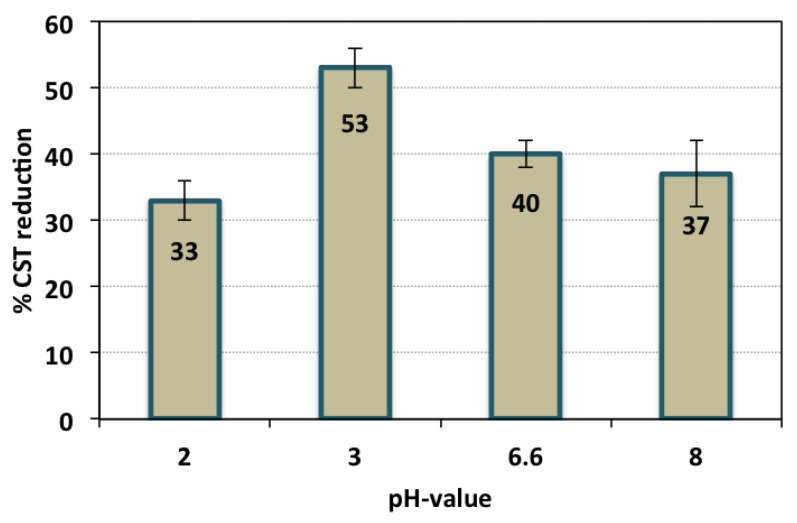
CST reduction efficiency at various pH values (operational conditions—conditioning time: 7 min; H_2_O_2_ dose: 800 mg/L; TAS-cake@Fe-(5-1) 1 g/L; and standard deviation of three replicates).

The influence of aluminum-based sludge dewaterability is exhibited in Figure 9, and the test was conducted by keeping the operating conditions constant at an H_2_O_2_ dose of 800 mg/L and TAS-cake@Fe-(5-1) 1 g/L. As given in the figure, the alum waste dewaterability performance was enhanced with the elevation of the pH value from 2.0 to 3.0 to record a 53% CST reduction. However, when increasing the pH condition to more than 3.0, the result is a reduction in the CST enhancement performance. This is due to the sludge particles’ surface presences, and characterization further defined the impact on the extracellular substance (TAS-cake@Fe-(5-1)) in the aluminum-based sludge. But, with acidic or alkaline circumstances for the alum sludge, the colloidal particles of the alum sludge might be hydrolyzed, and flocs might be formed. But, with the pH control and adjustment, alum sludge could adsorb both ions of H^+^ and/or OH^−^, thus influencing their self-chargeability. Moreover, it might be linked to the metal ion release from the sludge, i.e., aluminum and iron ions which could promote the flocculation, thus enhancing the water release from the aluminum-based sludge. This illustration was previously recognized by Kwon et al. [45] on wastewater sludge treatment and conditioning to enhance its dewaterability. Nevertheless, it is noted that higher pH results in a reduction in the quantity of OH radicals, which is believed to be the driving force towards improvements in sludge dewaterability. The effect of pH on the capillary suction time (CST) efficacy at a pH of 3.0 in this investigation is the best, corresponding to the highest net CST reduction efficacy being attained. Such results are in agreement with the previous findings of Lu et al. [48] and Tony [49] who claimed a similar efficiency of activated sludge dewaterability when exploring Fenton oxidation conditioning in an acidic pH range. Under the extreme acidic and alkaline medium, the structure of the extracellular sludge is reduced to small flocs [50,51].

#### 3.2.5. Effect of Temperature

**Figure 10:**
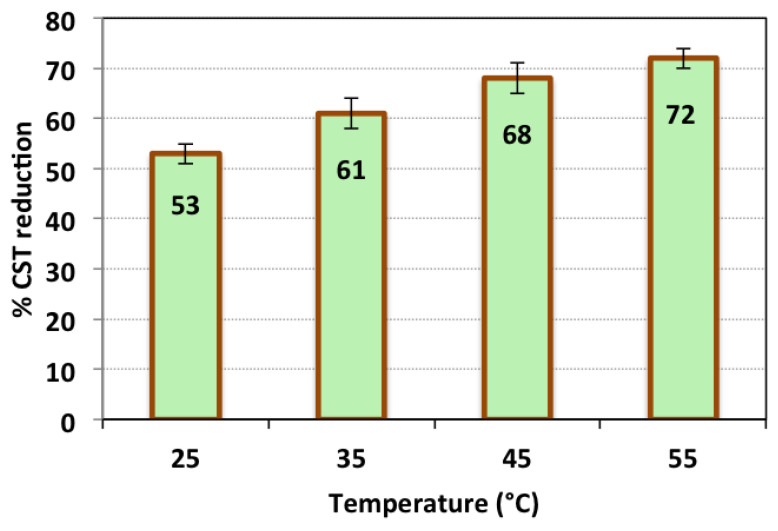
CST reduction efficiency at temperatures (operational conditions—conditioning time: 7 min; pH 3.0; H_2_O_2_ dose: 800 mg/L; TAS-cake@Fe-(5-1): 1 g/L; and standard deviation of three replicates).

Although the most common method for the Fenton reaction is conducted at ordinary room temperature, the temperature elevation could affect the conditioning system. In this regard, conditioning of the sludge is checked at various temperatures ranging from 25° to 55 °C. The test was conducted at operational conditions for a time of 7 min; pH 3.0; H_2_O_2_ dose of 800 mg/L; and TAS-cake@Fe-(5-1) 1 g/L. The results of alum sludge dewaterability are exhibited in Figure 10. The conditioning process was conducted using TAS-cake@Fe-(5-1): 1 g/Land H_2_O_2_ dose of 800 mg/L at pH 3.0. The results displayed in Figure 10 demonstrate that with the temperature elevation from room temperature to 55 °C, the sludge conditioning and dewatering performances were enhanced from a 53% CST reduction to 72%. Thus, the thermal effect has a positive influence on sludge conditioning. Temperature possesses a significant influence on the water release, thereby increasing the sludge dewatering rate. However, it is worth mentioning that, although the rate of hydrogen peroxide consumption is high at higher temperatures, the thermal effect with the Fenton reaction leads to a higher effect than solo Fenton oxidation [47]. It is noteworthy that the temperature elevation could enhance the rate of water release from the sludge molecules due to its thermal effect on the sludge particles. This leads to the dual conditioning of thermal and chemical dewatering [32]. This investigation is in accordance with the previous findings of Mustranta and Viikari [50], who described a suggestive enhancement in activated sludge conditioning performance with the temperature elevation.

#### 3.2.6. Comparison of Fenton with Commercial Conditioners

**Figure 11:**
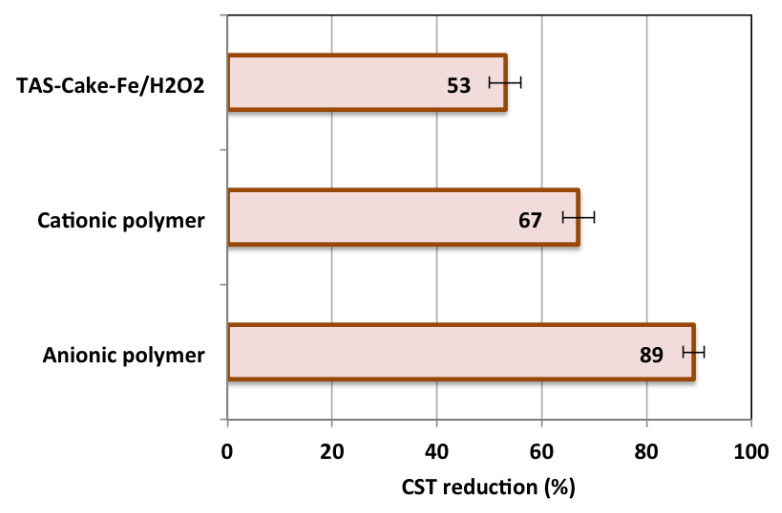
Comparison of various CST reduction efficiencies for different systems.

It is essential to compare the recently introduced sustainable facility for conditioning with the traditionally occurring commercial polymers. Figure 11 depicts typical results for the optimal TAS-cake@Fe-(5-1)-based Fenton system in comparison with polyelectrolyte conditioning. Initially, anionic and cationic polyelectrolytes were checked for their optimal time and doses (data are not shown). The optimal working time was recorded at 3 min of conditioning time, and the optimal polyelectrolyte was 10 mg/L for both polyelectrolyte types. The data in Figure 11 expose the consequence of the optimal conditioning circumstances for all conditioners. The results revealed that the highest conditioning performance was recorded for the anionic polyelectrolyte which revealed a CST reduction resulting in 89%. However, the cationic polyelectrolytes could condition the sludge to reach a 67% reduction in the CST. The polyelectrolyte presence improved the dry solid content in the sludge for floc formation. Notably, this was achieved because the efficacy of the conditioning in the case of the cationic polyelectrolyte differs, and a higher achievement was obtained compared to that of the anionic polymer. This is believed to be owing to the differences in the nature and the ionic charge of the polymers, which control the coagulation and flocculation [33].

In comparison to the TAS-cake@Fe-(5-1)-based Fenton system that could not bridge the high floc size of alum sludge molecules, the produced sludge flocs for polyelectrolyte were larger than that corresponding to its optimal TAS-cake@Fe-(5-1)-based Fenton conditioning; thereby, the water release was reduced in the TAS-cake@Fe-(5-1) system compared to polyelectrolytes. The result is a high CST reduction in polyelectrolytes compared to the Fenton system.

Although the lowest performance was associated with the TAS-cake@Fe-(5-1)-based Fenton system, it is noteworthy to mention that the sustainable TAS-cake@Fe-(5-1) catalyst is a waste-valorized material, and the magnetite is an environmentally non-hazardous material. Also, the toxicity of synthetic polyelectrolytes is still a concern for future uses and applications [37].

#### 3.2.7. Comparative Investigation

Comparing the current work with the dewatering data from the literature is essential to evaluate the current study performance. The CST filterability guide was assessed according to the optimum operational values and conditions. The comparative results displayed in Table 2 show the consequences of various sludge dewatering treatments using different conditioners. According to the data tabulated in Table 2, various forms of the Fenton reaction were categorized as significantly reasonable conditioners for various sludge types. Although various conditioning times are essential, the highest dewatering performance is not linked to the time period. Also, it was established that pronounced dewatering was achieved at 98% for the classical Fenton system. However, in the current study, it reached only 53%. But, it is essential to mention that the current study is based on introducing a recyclable material as a conditioning alternative rather than using fresh chemicals since the current catalyst is based on a recoverable waste material as well as magnetite in environmentally non-hazardous material. 

The Fenton oxidation application as a conditioner showed a promising superior dewatering technique that has numerous advantages. The use of Fenton oxidation in sludge minimizing is signified as a safe alternative for the application of polyelectrolyte as a sludge conditioner concerning the achievement of a more sustainable sludge management strategy. Moreover, when the catalyst source of the Fenton system is derived from a waste stream, especially sludge as a source of recyclable metals, this is a reliable way to minimize waste. Although it possesses a reduced tendency for dewatering rather than that of polymer conditioning or classical Fenton reagent conditioning, the suggestive catalyst from the waste stream offers the potential advantage of eliminating the perceived long-term risk of polymer residuals or introducing fresh chemicals to the environment. Thus, although the performance is less than that illustrated by classical Fenton, it is essential to note that this source of elements is from other sludge waste and magnetite. Thus, magnetite is signified as an environmentally non-hazardous material and is used in small proportions. Further, recovered sludge use is categorized as a win–win technology to attain the opportunity of reducing waste.

#### 3.2.8. Statistical Optimization

A Box–Behnken factorial design based on RSM (response surface methodology) was applied to optimize the combined operational parameter effects, namely TAS-cake@Fe-(5-1); H_2_O_2_ concentrations; and pH value. This is because the Fenton system is a multi-variable system (using SAS software, SAS (1990 Version)). The range variables are located as the preliminary optimized work and levels according to the design tabulated in Table 3. Further, 15 runs of the combined tests were, thereby, designed, and the design structure is shown in Table 3.

Primarily, the initial stage in the RSM is to expose an appropriate approximation between the response (CST reduction, %) and the set of independent variables. The polynomial second-order model for the three variables is correlated according to Equation (2).

(3) $$CST\left(\%\right)=78+5.42x_{1}-1.09x_{2}+5.00x_{3}-16.91x_{1}^{2}+0.90{\;x}_{1}x_{2}-0.50{\;x}_{1}x_{3}-9.95x_{2}^{2}-6.8\times 10^{-9}x_{2}x_{3}-11.03x_{3}^{2}$$

where *x*_1_, *x*_2_ and *x*_3_ are the coded independent variables, as tabulated in Table 3.

From the analysis of variance (ANOVA test, the data obtained from SAS demonstrated a good agreement between the experimental data and those predicted from the SAS analysis. The correlation was estimated and assessed from Fisher’s ‘*F*’ test, the probability value “*p*-values” and the regression coefficient “*R*^2^*”*. Generally, the model is reliable when the *p > F* is less than >0.05 (SAS, 1990), whereas *R*^2^ is more than 0.80 (SAS, 1990). Hence (SAS, 1990), the suggestive predicted model was checked for adequacy; the *p > F* value corresponded to 0.039, and the *R*^2^ value was 0.97. Those data verified the reliability of the model’s significance.

**Figure 12:**
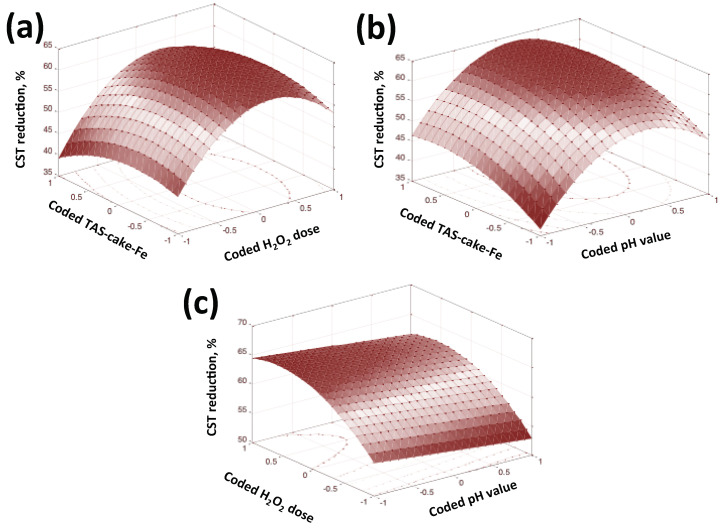
CST reduction (%) over surface and contour plots of (**a**) coded TAS-cake@Fe-(5-1) and H_2_O_2_; (**b**) coded TAS-cake@Fe-(5-1) and pH value; and (**c**) coded H_2_O_2_ and pH value.

For the object of model illustration, a graphical representation of such a model was established using the 3D surface and its corresponding 2D contour graphical illustrations (designed in MATLAB software version 7.11.0.584). The visualized representation in Figure 12 displays the response demonstrated as a CST reduction (%) that is enhanced (Figure 12a,b) by the increase in both TAS-cake@Fe-(5-1) and hydrogen peroxide. However, the suggestive CST improvement declined with the further increase in the reagents. Additionally, the decline in (%) CST occurred with high or low values of pH (Figure 12b,c). The plot confirms that the % CST reduction improved with the added Fenton reagent doses up to a specific limit, thus optimizing TAS-cake@Fe-(5-1) and H_2_O_2_ concentrations in addition to the pH value controlling the conditioning process.

To locate the optimum variables, Mathematical software (V 5.2. Wolfram Research Inc., Champaign, IL, USA) was utilized. The maximum values of the variables in their un-coded form were attained and recorded as follows: TAS-cake@Fe-(5-1) = 1.2 g/L; H_2_O_2_ = 740 mg/L; and pH value = 3.0.

Furthermore, to settle the model verification, an extra experiment with three replicates was conducted using the abovementioned optimum conditions. The results of such tests verified the optimal values that achieved a maximal XST reduction, reaching 54%, which clearly proved the efficacy of the suggested model.

## 4. Conclusions

The Fenton reagent conditioner based on TAS-cake@Fe-(5-1) augmented with H_2_O_2_ was applied to enhance the water release and sludge conditioning. The conditioner is based on the sustainability concept and uses a waste-recycled material, making it a green conditioner augmented with environmentally non-hazardous magnetite. The composite was investigated using XRD, FTIR and microstructure. The optimal working variables were used in optimized conditions, especially the higher yield that was based on the model optimization based on the RSM technique. The system parameters were investigated, and the optimal operating parameters were set as TAS-cake@Fe-(5-1) 1.2 g/L with H_2_O_2_ of 740 mg/L at pH 3.0, corresponding to a maximal CST reduction to 53%. Further data demonstrated that temperature elevation has a pronounced effect on the conditioning and dewatering performance, which reached 72% with the temperature elevation from room temperature to 55 °C. When 10 mg/L of both polyelectrolytes, anionic and cationic polymers, were used as conditioners, the CST reduction reached 89% and 67%, respectively. Thus, comparing Fenton oxidation with the polyelectrolytes as a commercial conditioner, the CST is less than attained by the polyelectrolytes. It is notable that the toxicity concern when polyelectrolytes were applied demonstrated the importance of the current study. Overall, this investigation focused on the circular economy approach.

## Figures and Tables

**Table 1:** Peaks and corresponding adsorption bands of FTIR peaks for TAS-cake@Fe-(5-1) composite conditioner.

Peak	Wavenumber (cm^−1^)
Fe–O bond	420 and 556
O–Si–O bending vibration	467.65
Al–Mg–OH bending vibration	1201.5
C=O bending	1420.32
C=C	1629
O–H stretching vibration	3440.38

**Table 2:** Fenton conditioning with various systems from previous studies compared with the current work.

Fenton Conditioner	Sludge	Time Required, min	Operational Circumstances *	Performance, %	Ref.
TAS-cake@Fe-(5-1)/H_2_O_2_	Aluminum-based sludge	7	TAS-cake@Fe-(5-1) (101 mg/g-DS); H_2_O_2_ (62 mg/g-DS); pH 3.0	53%	Current study
FeSO_4_/H_2_O_2_	Wastewater sludge	63	FeSO_4_.7H_2_O (80 mg/g-DS); H_2_O_2_ (20 mg/g-DS); pH 5.0	85%	[52]
Fe^2+^/H_2_O_2_	Wastewater sludge	120	Fe^2+^ (36 mM); H_2_O_2_ (360mM)	98%	[53]
Fe^2+^/H_2_O_2_	Wastewater sludge	30	Fe^2+^ (0.1 g/g-DS), pH 5.8,	79%	[20]
Fe^2+^/H_2_O_2_	Wastewater sludge	60	Fe^2+^ (750 mg/g-DS); H_2_O_2_ (825 mg/g-DS); pH 2.0	76%	[54]
Fe^2+^/H_2_O_2_	Municipal sludge	60	Fe^2+^ (32 mg/g-DS); H_2_O_2_ (40 mg/g-DS); pH 3.0	70%	[46]
Electro-Fenton	Industrial sludge	60	Fe^2+^ (15 mg/g-DS); H_2_O_2_ (25 mg/g DS)	94%	[55]
Fenton/Lime	Sewage sludge	90	Fe^2+^ (50 mg/g-DS); H_2_O_2_ (30 mg/g DS); pH 3.0; lime (50 mg/g DS)	96%	[43]
Fenton/Lime	Municipal sludge	120	Fe^2+^ (50 mg/g DS), H_2_O_2_ (30 mg/g DS); pH 3.0; lime (50 mg/g DS)	95%	[56]

* DS: dry solids.

**Table 3:** RSM factorial design in coded and un-coded levels for TAS-cake@Fe-(5-1)/H_2_O_2_ system.

Exp. no.	Variables					
	TAS-cake@Fe-(5-1) Dos		H_2_O_2_ Dose		pH-Value	
	Coded	Un-Coded	Coded	Un-Coded	Coded	Un-Coded
1	−1	0.5	−1	700	0	3.0
2	−1	0.5	1	900	0	3.0
3	1	1.5	−1	700	0	3.0
4	1	1.5	1	900	0	3.0
5	0	1.0	−1	700	−1	2.5
6	0	1.0	−1	700	1	3.5
7	0	1.0	1	900	−1	2.5
8	0	1.0	1	900	1	3.5
9	−1	0.5	0	800	−1	2.5
10	1	1.5	0	800	−1	2.5
11	−1	0.5	0	800	1	3.5
12	1	1.5	0	800	1	3.5
13	0	1.0	0	800	0	3.0
14	0	1.0	0	800	0	3.0
15	0	1.0	0	800	0	3.0

## Data Availability

Data are available upon request.
